# Cerebellar Transcranial Direct Current Stimulation in Children with Autism Spectrum Disorder: A Pilot Study on Efficacy, Feasibility, Safety, and Unexpected Outcomes in Tic Disorder and Epilepsy

**DOI:** 10.3390/jcm11010143

**Published:** 2021-12-28

**Authors:** Giordano D’Urso, Elena Toscano, Veronica Sanges, Anne Sauvaget, Christine E. Sheffer, Maria Pia Riccio, Roberta Ferrucci, Felice Iasevoli, Alberto Priori, Carmela Bravaccio, Andrea de Bartolomeis

**Affiliations:** 1 Unit of Psychiatry, Department of Neurosciences, Reproductive and Odontostomatological Sciences, University of Naples Federico II, 80131 Naples, Italy; elena.toscano@studio.unibo.it (E.T.); veronica.sanges5@hotmail.it (V.S.); felice.iasevoli@unina.it (F.I.); adebarto@unina.it (A.d.B.); 2 E.A. 4334 Movement, Interactions, Performance (MIP), CHU Nantes, University of Nantes, 44322 Nantes, France; Anne.SAUVAGET@chu-nantes.fr; 3 Department of Health Behavior, Roswell Park Comprehensive Cancer Center, Buffalo, NY 14203, USA; Christine.Sheffer@RoswellPark.org; 4 Department of Medical and Translational Sciences, Child and Adolescent Neuropsychiatry, University of Naples Federico II, 80131 Naples, Italy; piariccio@gmail.com (M.P.R.); carmela.bravaccio@unina.it (C.B.); 5 Aldo Ravelli Research Center for Neurotechnology and Experimental Brain Therapeutics, Department of Health Science, University of Milan, Via A. di Rudinì, 8, 20142 Milan, Italy; roberta.ferrucci@unimi.it (R.F.); alberto.priori@unimi.it (A.P.); 6 ASST Santi Paolo e Carlo, San Paolo University Hospital, Via A. di Rudinì, 8, 20142 Milan, Italy

**Keywords:** tDCS, cerebellum, autism spectrum disorder (ASD), epilepsy, tic disorder

## Abstract

Patients with autism spectrum disorder (ASD) display distinctive neurophysiological characteristics associated with significant cognitive, emotional, and behavioral symptoms. Transcranial direct current stimulation (tDCS) applied to the frontal or temporoparietal lobes has demonstrated potential to reduce the severity of ASD-related symptoms. Recently, the cerebellum has been identified as a brain area involved in ASD pathophysiology. In this open-label pilot study, seven ASD patients aged between 9 and 13 years underwent 20 daily sessions of 20 min cathodal stimulation of the right cerebellar lobe. At the end of the treatment, the Aberrant Behavior Checklist (ABC) scores showed a 25% mean reduction in global severity of symptoms, with a more pronounced reduction in the “social withdrawal and lethargy” (−35%), “hyperactivity and noncompliance” (−26%), and “irritability, agitation, and crying” (−25%) subscales. Minor and no improvement were observed in the “stereotypic behavior” (−18%) and “inappropriate speech” (−0%) subscales, respectively. Improvements were not detected in the two patients who were taking psychotropic drugs during the study. Clinical response showed a symptom-specific time course. Quality of sleep and mood improved earlier than hyperactivity and social withdrawal. The treatment was generally accepted by patients and well tolerated. No serious adverse events were reported. Stimulation also appeared to markedly reduce the severity of tics in a patient with comorbid tic disorder and led to the disappearance of a frontal epileptogenic focus in another patient with a history of seizures. In conclusion, cerebellar tDCS is safe, feasible, and potentially effective in the treatment of ASD symptoms among children. Strategies to improve recruitment and retention are discussed.

## 1. Introduction

Autism spectrum disorder (ASD) is a complex neurodevelopmental disorder, the prevalence of which has increased significantly in the past 20 years [1]. ASD is characterized by social impairment, difficulties in language and communication, stereotyped behaviors, and restricted or repetitive interests. ASD is commonly associated with multiple additional abnormalities, including intellectual disability, epilepsy, somatosensory abnormalities, sleep disturbances, and gastrointestinal symptoms [2]. As a result, the quality of life and the global functioning of children and adults with ASD are severely compromised, causing significant emotional suffering among those diagnosed and their families [3,4].

Even though genetic and environmental factors appear to make significant contributions to the development of ASD [5,6], the exact etiology and pathophysiology are still unclear [7]. In morphometric brain imaging studies, compared to neurotypical children, children with ASD showed hemispheric asymmetry with greater volume in several right hemispheric structures compared with homologous contralateral structures, particularly in brain regions associated with language and social abilities [8,9,10,11]. In addition, a functional magnetic resonance imaging study found hypoactivation of specific brain areas of the left hemisphere (i.e., amygdala and fusiform gyrus) relative to the right hemisphere among children with ASD compared with neurotypical children [12], while a proton magnetic resonance spectroscopic study showed a reduced N-acetylaspartate/creatine/phosphocreatine ratio, a marker of neuronal density, in the left but not in the right dorsolateral prefrontal cortex (DLPFC) of autistic patients [13]. Moreover, impairment of the intracortical inhibitory function (excitatory/inhibitory (E/I) imbalance) in the left DPFC might contribute to the pathogenesis of autism, accounting for several motor, sensory, and cognitive features of the disorder [13]. The left DLPFC is key to cognitive, social, and emotional functioning [14] and is essential for the theory of mind (ToM), the recognition of emotions, and executive functions, all of which are typically impaired in autistic individuals [13]. Finally, some individuals with ASD show a progressive developmental disorder in the brain with aberrant decline of cortical plasticity [15], abnormal cytoarchitectural maturation [16], impaired brain connectivity [17], and mirror neuron dysfunction [18]. Because the high-level skills that are commonly impaired in ASD require efficient integration of multiple short- and long-range circuits, ASD probably should not be attributed to the dysfunction of single brain areas or circuits but instead to the breakdown of multiple integrated short- and long-range circuits.

Congruent with this broad perspective, the role of the cerebellum is now attracting considerable interest. Recent evidence suggests that individuals with ASD demonstrate noncanonical connectivity between prefrontal and cerebellar cortices [19,20]. The proper functioning of cerebrocerebellar loops is extremely important for early cortical development. Theoretically, disruptions of these loops in ASD might impede the specialization of cortical regions involved in motor control, language, and social interaction and account for the impairments of these functions [21]. In particular, functional connectivity (FC) studies have demonstrated that autistic children show increased FC between nonmotor areas of the right cerebellar hemisphere (lobule VI and Crus I) and specific regions of the homolateral cerebral cortex (primary sensory, premotor/primary motor, and occipital cortices), violating the typical patterns of contralateral cerebrocerebellar connectivity [22,23]. This increased functional connectivity between unexpected, noncanonical regions might occur at the expense of canonical connectivity between regions involved in language and social interaction. In fact, compared to their neurotypical counterparts, autistic children and adolescents display reduced FC between right posterior cerebellum and contralateral prefrontal regions, such as the dorsolateral and medial prefrontal cortex. These alterations in cerebrocerebellar FC are correlated with greater ASD symptom severity [23].

Unfortunately, at present, there is no cure for autism. Patient symptomology and treatment goals often vary enormously, and interventions often include an individualized combination of behavioral and pharmacological interventions [24,25]. These interventions have shown some positive results in many individuals with ASD, but they rarely lead to significant improvements in the core symptoms of autism, i.e., deficits in social interactions and communication as well as restricted and repetitive interests and activities. New, accessible, and more effective treatment options are urgently needed. In recent years, there has been growing interest in the therapeutic potential of noninvasive brain stimulation techniques (NIBS), such as repetitive transcranial magnetic stimulation (rTMS) and transcranial direct current stimulation (tDCS), as novel treatments for several psychiatric and neurological disorders [26,27,28]. The use of NIBS in patients with psychiatric disorders is rapidly making its way from research settings to clinical practice and is leading to the establishment of psychiatric units entirely dedicated to neuromodulation [29]. In particular, tDCS is a noninvasive, safe, painless, and easy-to-use procedure for the focal modulation of cortical brain activity that can be administered with negligible side effects [30]. tDCS involves the application of a weak electrical current (1–2 mA) to specific areas of the brain via one or more electrodes placed on the scalp. By convention, it is assumed that the current enters the head from the anode (positive electrode) and exits out the cathode (negative electrode). The current path and density depend on the position of electrodes and the characteristics of the interposed structures, with low impedance compartments favoring the current passage. The orientation of neuronal fibers has also been reported to influence the current direction [31]. TDCS alters the resting potential of the neuronal membranes in the cortex underlying the area of the electrodes. In particular, anodal tDCS decreases the resting membrane potential, making the neurons more likely to fire an action potential, while an opposite effect is observed under the area of the cathode [32]. Several concurrent mechanisms have been proposed for this change in membrane potential, including local changes in cerebrospinal fluid pH and ion concentrations as well as migration of and allosteric changes to neuronal membrane proteins [33]. Even if the change in membrane potential induced by anodal stimulation is not sufficient to reach the firing threshold, long-term potentiation (LTP)-like effects have been observed. Similarly, long-term depression (LTD)-like effects have been observed following cathodal stimulation. Nonetheless, the overall results are reflected in changes to excitability and spontaneous firing rates across the stimulated areas [34,35] as well as the trans-synaptic activation of interconnected remote regions [36].

The therapeutic effects of tDCS are linked to polarity-dependent neurophysiological changes induced in the target cortical areas, including increases (by anodal stimulation) or decreases (by cathodal stimulation) in cortical excitability [37,38]. Anodal tDCS has received far more attention in research on the clinical applications of tDCS, with the active excitatory electrode being located over the left DLPFC in the majority of trials [33]. The placement of the cathode varies considerably between studies, and trials assessing the specific effect of cathodal stimulation are rare [39]. One possible reason for this might be that the neurophysiological effects of cathodal stimulation are less consistent than those of anodal stimulation, and researchers are thus more cautious in applying protocols with cathodal stimulation. In fact, reductions in excitability can result in facilitation when cathodal tDCS is administered under certain circumstances (e.g., under the effect of serotonergic drugs) [23]. Moreover, while the physiological and behavioral effects of cathodal-inhibitory tDCS are sufficiently established in the sensory and motor domains, they are less clearly established for higher-order brain regions and functions [40].

tDCS is being increasingly used in the treatment of several psychiatric disorders [28], including depression [41], obsessive compulsive disorder [42,43], anxiety disorders [44], substance use disorders [45], and schizophrenia [46]. tDCS can be used alone or in combination with other interventions, such as psychotherapy [47] or cognitive training [48]. In recent years, tDCS has also been used to treat children and adults with ASD, with no serious adverse events and encouraging results in terms of feasibility, efficacy, and tolerability [49,50]. Of particular interest, preliminary studies have shown tDCS to improve the core symptoms of ASD in addition to reducing behavioral symptoms [51]. We speculate that tDCS might achieve these therapeutic benefits by acting on specific pathophysiological mechanisms of ASD.

We chose to position the cathode, the negative inhibitory tDCS electrode, on the cerebellum to address potential deficits in inhibitory neural function in the cerebellum among individuals with ASD. This rationale is consistent with the rationale applied in two previous studies from our group in which we targeted the left DLPFC to address potential deficits in inhibitory neural function. In these studies, the cathodal stimulation of left DLPFC with an extracephalic reference led to beneficial behavioral effects in adults with ASD [52,53].

Among individuals with ASD, there is a significant decrease in the number of Purkinje cells, the most prevalent inhibitory neuron in the cerebellum [54]. These cells have an important role in the physiological regulation of the contralateral transthalamic projections to the neocortex [55]. In addition to the numerical reduction in Purkinje cells, other indications are associated with defective inhibitory function in the cerebellum and excitatory/inhibitory (E/I) imbalance in individuals with ASD [56], namely higher mean glutamate levels, reduced glutamate reuptake, increased mRNA levels of AMPA and NMDA glutamate receptors with reduction in their respective receptor densities [57,58], lower levels of GABA-A receptor units [58], and alteration of protein expression in glutamatergic and GABAergic neurons [59]. The numerical reduction in Purkinje cells and the glutamate/GABA imbalance contribute to impaired modulatory output from the cerebellar hemispheres and altered cerebrocerebellar feedback loops [60], as also shown by mouse models of cerebellar degeneration [61]. Among individuals with ASD, low cerebrocerebellar connectivity and altered glutamate/GABA balance co-occur, likely accounting for some of their cognitive abnormalities [60]. Cathodal tDCS has been proposed to restore E/I balance toward a more typical level in targeted regions in ADHD patients [62].

Given this evidence, we hypothesized that cathodal inhibitory tDCS might compensate for the defective inhibitory function reported in the cerebellum of individuals with ASD, restore E/I balance in that region, and improve cerebrocerebellar communication. This open-label, proof-of-concept pilot study aimed to conduct a preliminary investigation of the therapeutic effects and feasibility of this novel tDCS protocol in a sample of children with ASD. The primary outcome was the reduction of ASD-related aberrant behaviors. Secondary outcomes included feasibility, safety, and potential unexpected effects of this tDCS protocol.

## 2. Materials and Methods

### 2.1. Participants

Children aged 6–17 with a confirmed diagnosis of ASD were included. The exclusion criteria included skull defects, presence of metallic elements or implantable devices in the head, and any severe medical condition that might interfere with study procedures. Participants were recruited by offering enrollment to all consecutive new patients of the ASD outpatient clinic of the Unit of Child and Adolescent Neuropsychiatry of the University of Naples “Federico II” who met the age criterion during a one-month period. We enrolled 7 children aged 9 to 13 years (mean age ± SD: 11 ± 1.15) consisting of 6 males and 1 female. Table 1 presents the demographic and clinical characteristics of the participants.

### 2.2. Assessment

We used the Autism Diagnostic Observation Schedule, Second Edition (ADOS-2) to confirm the diagnosis of ASD. The ADOS-2 is the “gold standard” for observational assessment of ASD and consists of a semistructured set of observations and a series of play-based activities that involve the participant and a trained clinician. These activities are designed to obtain information about communication skills, social interaction, and imaginative use of materials. The ADOS-2 has four separate modules, with each module aiming at a specific level of expressive language ability. By observing and coding the elicited behaviors, information can be attained for diagnosis and treatment planning [63].

To detect the effects of tDCS, the Aberrant Behavior Checklist (ABC) was administered to the caregivers, who in this case were the parents for all patients, by a trained psychologist at baseline and 1 week after the completion of treatment. This checklist was specifically developed to identify the effects of therapeutic interventions on behavioral disturbances of individuals with developmental disorders. The ABC consists of 58 items scored from 0 (‘‘not at all a problem’’) to 3 (‘‘problem is severe in degree’’). It has an overall score and five subscale scores corresponding to different symptom domains: (1) irritability, agitation, and crying, (2) lethargy/social withdrawal, (3) stereotypic behavior, (4) hyperactivity/noncompliance, and (5) inappropriate speech [64].

To detect more subtle and time-dependent effects, we prepared a self-rated observation sheet to be completed by caregivers of the participants with the assistance of a research team member. At baseline, caregivers were asked to indicate the most disturbing and disabling behavioral problems in the participant’s everyday life. We then selected the problems shared by all and asked the caregivers to rate the change in the severity of those symptoms, if present, on a daily basis while participants were undergoing tDCS. We used a bipolar numeric visual analog scale (VAS) [65]. Changes in symptom severity were assessed on a 10-point VAS, with 0 corresponding to “very much improved/symptom absent”, 5 corresponding to “symptom unchanged”, and 10 corresponding to “very much worsened/worst-ever severity of the symptom”. This scoring method was chosen to maximize sensitivity to tDCS-induced changes, regardless of pre-existing symptom severity [66].

To assess feasibility, we employed two measures: (1) the acceptance rate, i.e., the percentage of eligible subjects that accepted participation during the specified recruitment time period and (2) the retention rate, i.e., the percentage of included subjects that completed the 20-session/four-week treatment protocol [67].

We did not use standardized instruments to assess the occurrence of side effects and adverse events. To this end, we relied on direct observation, daily interviews with the patients’ caregivers, and on an open-ended question at the end of the self-rated observation sheet described above. This open-ended question was aimed at detecting any unexpected consequence of treatment.

For the one participant with a severe tic disorder (Pt 1), we also used the Yale Global Tic Severity Scale (YGTSS) administered before and after tDCS. For the one participant with epilepsy (Pt 7), we obtained an EEG recording at baseline and another 1 week after tDCS.

### 2.3. tDCS

All participants underwent daily sessions of 1.0 mA 20 min tDCS on 20 consecutive weekdays [50,68]. We used a HDC stimulator (Newronika^TM^, Milan, Italy) connected to two 5 cm × 5 cm conductive-rubber electrodes. Each electrode was covered with conductive gel and enclosed in a spongy pocket, which in turn was soaked with equal amounts of saline solution and tap water. The cathode was placed over the right cerebellar hemisphere, 1 cm below and 4 cm lateral to the inion (corresponding to the cerebellar lobule VII on the scalp), and the anode was placed over the F3 position according to the international 10–20 EEG system (corresponding to the left DLPFC). The F3 position was located according to Beam et al., 2009 [69] using a measuring tape and a medical skin marker. The electrodes were secured to participants’ heads by means of a 10 cm piece of tubular elastic net for newborn umbilical dressing. During the stimulation, subjects were watching emotionally neutral animation videos. Each application was carried out with constant operator supervision. During the period that participants were receiving tDCS, no changes were made to their ongoing pharmacological and behavioral therapies.

### 2.4. Statistics

ABC scores were analyzed using the statistical functions of Microsoft Office Excel 2007. Results are expressed as means ± SD for continuous variables. Variations of measured parameters were defined by subtracting the baseline scores from those reported after tDCS. Negative values therefore indicated improvement. The percentage change from baseline was also calculated for each scale/subscale mean value. For the assessment of within-subject changes after tDCS, two-tailed paired *t*-tests were computed for the mean total scores of the ABC scale and for each of the five subscale scores. Considering the small sample size and the consequent risk of type 1 errors, no threshold for statistical significance was established.

### 2.5. Ethical Factors

All procedures in this study were performed in accordance with the Declaration of Helsinki and approved by the Ethics Committee for the Biomedical Activities of the University of Naples “Federico II” (Ethics Committee protocol 61/10). All the parents of the study participants, and the patients themselves when possible, signed the informed consent form approved by the Ethics Committee.

## 3. Results

Seven out of 12 eligible participants were enrolled (acceptance rate = 58.3%). The parents of five patients who did not accept reported that they were unable to comply with the study requirements for practical issues (living out of the Naples area and/or organizational problems of the family). All participants completed all scheduled tDCS sessions (i.e., four weeks/20 sessions; retention rate = 100%).

**Figure 1:**
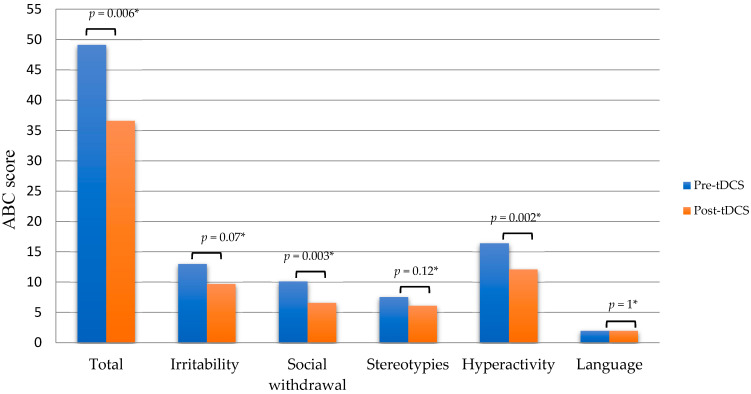
Aberrant Behavior Checklist mean scores before (pre-tDCS) and after (post-tDCS) transcranial direct current stimulation treatment. * Due to the low statistical power of this pilot study, no significance threshold was established, and *p*-values should be considered cautiously.

The observation and the coding of the behaviors elicited by ADOS-2, administered by a child psychiatrist experienced in the diagnosis of ASD (MPR), confirmed that all participants fulfilled DSM-V criteria for ASD. At baseline, the ABC total scores ranged from 15 to 97 (mean 49.14 ± 26.77), whereas the scores ranged from 13 to 75 (mean 36.57 ± 21.74) after tDCS, a mean difference of −12.57 ± 34.5 (−25%, *p* = 0.006). Similar changes were found in the five ABC subscales, with subscale 1 (irritability, agitation, and crying) showing a mean difference of −3.28 ± 11.85 (−25%; *p* = 0.07) from the baseline value of 13 ± 9.72, subscale 2 (social withdrawal and lethargy) showing a mean difference of −3.57 ± 5.48 (−35%; *p* = 0.003) from the baseline value of 10.14 ± 4.48, subscale 3 (stereotypic behavior) showing a mean difference of −1.42 ± 5.98 (−18%; *p* = 0.12) from the baseline value of 7.57 ± 4.89, subscale 4 (hyperactivity and noncompliance) showing a mean difference of −4.28 ± 15.06 (−26%; *p* = 0.002) from the baseline value of 16.42 ± 10.95, and subscale 5 (inappropriate speech) showing a mean difference of 0 ± 3.1 from the baseline value of 2 ± 2.23 (−0%; *p* = 1). Because this design is very likely to be underpowered, all *p*-values should be considered cautiously (see Figure 1).

**Figure 2:**
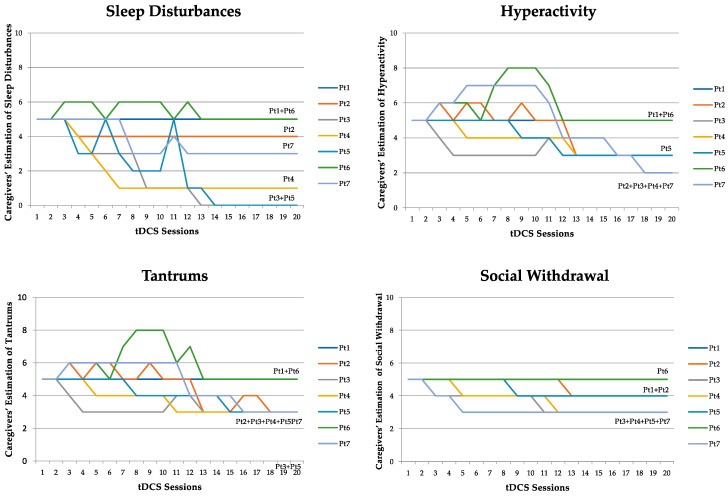
The four graphs display the severity rating of the four behavioral symptoms indicated as the most disturbing by the patients’ caregivers, throughout the 20 daily tDCS sessions. One stripe in a graph corresponds to one patient. The same patient is represented with the same color in all four graphs. In the y-axis, 5 corresponds to “symptom unchanged”, 0 corresponds to “very much improved/symptom absent”, and 10 corresponds to “very much worsened/worst-ever severity of the symptom”. Pt = patient.

According to the self-rated observation sheets, three out of the four most disabling problems identified at baseline by the parents coincided with those assessed by the ABC, i.e., hyperactivity, tantrums, and social withdrawal (corresponding to the subscales hyperactivity/noncompliance; irritability, agitation, and crying; and lethargy/social withdrawal of the ABC, respectively). However, sleep disturbances were also identified as extremely important to quality of life and overall functioning. The daily reports revealed that symptom improvement was present in all participants who were not receiving pharmacotherapy. Participants receiving pharmacotherapy either did not respond (Pt 1) or responded with different patterns (Pt 6). The time course of clinical change appeared to be symptom-specific, with “sleep disturbances” improving appreciably from the first week of tDCS, while hyperactivity, tantrums, and social withdrawal improved from the second or third week onward. In the first two weeks, improvements faded after the break in sessions during the weekend. However, after the third week, improvements appeared to be maintained during the weekends (see Figure 2).

No serious adverse events were observed or reported. Three patients showed or reported a mild, temporary skin irritation at the site of stimulation. Pt 1, who suffered from a severe tic disorder, showed a 90% reduction in the YGTSS total score after completing the tDCS sessions. This improvement was maintained during the three-month follow-up. Pt 7 had two epileptic foci in the baseline EEG, with one in the left frontal area and one in the left temporal area. The EEG recorded one week after the end of tDCS sessions no longer showed the focus in the frontal area.

## 4. Discussion

These findings represent the first report of individuals with ASD undergoing tDCS targeting the cerebellum. Cathodal tDCS over the right cerebellar lobe led to an improvement of the ASD symptoms among all participants. The mean ABC score was reduced by 25% on average. The most pronounced improvements were seen in the “social withdrawal and lethargy” (−35%; *p* = 0.003), “hyperactivity and noncompliance” (−26%; *p* = 0.002), and “irritability, agitation, and crying” (−25%; *p* = 0.07) subscales of the ABC. Minor or no improvement was observed in the “stereotypic behavior” (−18%; *p* = 0.12) and “inappropriate speech” (−0%; *p* = 1) subscales. Although the *p*-values reported should be considered very cautiously as the study is underpowered and the confidence intervals are very large due to the sample heterogeneity, it remains noteworthy that all seven participants showed considerable improvement. Moreover, improvements were confirmed with two sources of information, namely clinical observation and the caregivers’ daily reports.

We speculate that these findings might be due to multiple factors, including the restoration of intracerebellar inhibition, which in turn might have improved cerebrocerebellar loops, whose alterations are correlated with greater ASD symptom severity [23]. The rationale for targeting the cerebellum was described in detail above (see Introduction). Considering the beneficial effect of our tDCS protocol, we can hypothesize that the stimulation might have enhanced the contralateral canonical connectivity between the right cerebellum and the left frontal lobe, involved in language and social interaction, which was found to be reduced in autistic children compared to their neurotypical counterparts [23]. This enhancement could have occurred at the expense of the noncanonical homolateral connectivity, which was found increased in autistic patients [22,23].

However, the reference electrode, i.e., the anode placed in correspondence of the left DLPFC, likely also had an important role in producing the clinical effects. Proton magnetic resonance spectroscopy studies of individuals with ASD showed lower levels of N-acetyl aspartate (NAA), a marker of mitochondrial function and neural density, in the left DLPFC [13]. This evidence, along with the hypothesis of an intracortical inhibitory dysfunction and relative hypoactivity of the left hemisphere compared to the right, suggests that anodal tDCS over the left DLPFC could have improved ASD-related symptoms through its effects on cognitive processes associated with left DLPFC activity, such as attention, memory, and social functioning.

These findings are consistent with those reported by Amatachaya and colleagues (2014) from a randomized double-blind placebo-controlled trial of anodal (excitatory) or sham tDCS over the left DLPFC of 20 children with ASD for five consecutive days. They reported significantly more improvement among participants who received active tDCS compared to sham in all symptom domains, except for language [70]. Nonetheless, those findings are not directly comparable to the findings in our study for methodological reasons. The Amatachaya et al. sample was younger than ours (mean age 6.4 vs. 11), the rating scales were different, and the cathode was placed over the right shoulder instead of the cerebellum, probably accounting for a very different direction in the current. Regarding this latter aspect, in our protocol, the current interconnected the right cerebellar and left frontal areas, a connection which is physiologically activated during social paradigms (e.g., abstract mentalizing), imitation, emotional face, and biological motion processing [71,72]. In the light of studies showing that the therapeutical effect of brain stimulation on psychiatric symptoms is accompanied by FC changes in illness-specific circuitry [73], we can hypothesize that our protocol, which was different from the one by Amatachaya et al., could have specifically acted on the above connection and its related behavioral implications.

Of note, the combination of the ABC results and the information gathered through the self-observation sheets indicated a substantial difference in response among participants receiving pharmacotherapy in this study. This might be due to the action of the medications interfering with the mechanisms by which tDCS achieves its effects [23,74]. Pt 6 was receiving antipsychotic medications while undergoing tDCS (haloperidol 1 mg/qd and risperidone 5 mg/qd). Previous studies have demonstrated that the dopaminergic system has a critical role in the neuromodulatory effect of tDCS [75] via a nonlinear association between dopamine receptor activity and the tDCS effect, which can be blocked either by over- or underactivation of D1- or D2-like receptors [76]. Haloperidol is an antipsychotic with high D2 affinity and is known to suppress tDCS-induced plasticity [76]. This could explain why Pt 6 showed a different response to tDCS and obtained an overall poorer outcome. This observation, if confirmed by future studies, has clinical implications in terms of selecting individuals with ASD who will benefit from tDCS treatment. Pt 1, the other patient receiving pharmacotherapy during the tDCS treatment, was taking sertraline (100 mg/qd) prescribed for tic disorder. Although he demonstrated significant improvement in the symptoms of tic disorder following the tDCS course, he showed no improvement in any of the four ASD symptom dimensions. Other studies have found antidepressants to increase the effect of tDCS among individuals with depression [77]. The interpretation of our findings might be that these participants had more severe ASD or a type of ASD with different etiology or pathophysiology.

The chronology of the clinical effects of tDCS is a novel observation and also quite interesting. Improvements in the quality of sleep occurred prior to improvements in hyperactivity, tantrums, and social withdrawal. We hypothesize that the early-onset improvements were due to the effect of tDCS on the directly stimulated areas or to an aspecific “calming” effect of the current, while delayed improvements were likely due to subsequent and more complex modification of disease-specific abnormal circuitry, such as frontocerebellar networks. The improvement of sleep induced by tDCS was the effect most appreciated by the parents. At baseline, parents reported that sleep disturbances were among the most distressing behavioral problems, frequently affecting the quality of life of the entire family. Interestingly, tDCS has been used to enhance sleep in young students/athletes and in the elderly, with the aim of improving their performance [78,79].

Indeed, prefrontal and cerebellar regions have been implicated in sleep processes [80], and sleep modulation represents one of the nonmotor functions of cerebellum [81]. A study of adult euthymic bipolar patients also showed that prefrontal–cerebellar tDCS, with the cathode over the right cerebellum and the anode over the left DLPFC, improved sleep quality [82]. The approach used in this study was very similar to that of the present trial with the exception of the exact position of the electrode used to target the left DLPFC (Fp1 instead of F3, according to the 10–20 EEG system) and for the number of tDCS sessions (15 sessions in three weeks instead of 20 sessions in four weeks). The current intensity of 2 mA is comparable to 1 mA used in the current study when taking into account the difference in head anatomy between adults and children [68]. Thus, we can argue that the concomitant cathodal stimulation of the cerebellum and anodal stimulation of the left DLPFC may specifically modulate functionally impaired sleep-related networks among individuals with ASD, thus improving sleep quality.

The stabilization of the tDCS-induced clinical effects from the third week onward, despite the absence of tDCS sessions during the weekends, might reflect a cumulative effect, which supports the potential persistence of improvements once the tDCS sessions are discontinued.

The recruitment rate was 58.3%, with 5 out of 12 eligible patients refusing to participate in the study for reasons related to ability to attend all the sessions due to travel and/or the burden of daily attendance on family routine. To mitigate this challenge, we suggest future studies be carried out in multiple local facilities as close as possible to the patients’ homes, ideally in rehabilitation centers where patients already receive treatment. In addition, the impact of reducing the number and/or frequency of tDCS sessions should be explored. The retention rate was a remarkable 100%, with all participants completing the entire study protocol. These findings indicate that this approach is well accepted and that when patients and caregivers commit, they are likely to complete the recommended course.

Unexpectedly, we found two important clinical outcomes. Pt 1, with comorbid tic disorder, showed a 90% improvement of frequency and intensity of tics, and this improvement remained stable through the three-month follow-up visit. ASD and tics share common pathophysiology (i.e., E/I imbalance in corticostriatal circuits). The tics in Tourette syndrome are associated with dysfunction of the striatal GABAergic inhibitory loops, leading to an excess of striatal dopamine [83]. Dopamine, in turn, causes an abnormal functioning of the basal ganglia–thalamocortical motor network, leading to abnormal movements. Furthermore, basal ganglia, which have a role in triggering movements, are linked with the cerebellum, which have a role in motor pattern amplification according to recent thinking. Basal ganglia influence cerebellar activity through the subthalamic–pons–cerebellar–disynaptic link [84]. We speculate that the cathodal inhibitory stimulation in this instance reduced activity of the cerebellum and modulated one of the areas involved in tic production [85].

The second unexpected finding involved Pt 7, who was diagnosed with epilepsy in the first year of life. In the baseline EEG, he had two epileptic foci, one in the left frontal area and one in the left temporal area. One week after the end of tDCS, the focus in the frontal area was no longer detectable. Several studies to date have investigated the role of tDCS in the treatment of epilepsy. Highly heterogeneous methods have likely contributed to inconclusive results overall [86]. However, none of those previous trials involved tDCS of the cerebellum, even though preclinical studies of deep brain stimulation have reported that cerebellar stimulation might control epileptogenesis [87]. Recent evidence has highlighted the important role of the cerebellum in controlling epileptic seizures and particularly epileptic discharges in frontal lobe epilepsy [88]. Of note, frontal lobe epilepsy is usually accompanied by dysfunctional connectivity between the frontal lobe and cerebellum [89], a dysfunction also found in nonepileptic autistic patients [72]. We can speculate that in Pt 7, cathodal tDCS of the cerebellum might have modified the intracerebellar E/I balance and subsequently improved cerebellar brain communication [62] and that this might have contributed to the disappearance of the frontal epileptogenic focus. Considering the high prevalence of epilepsy among individuals with ASD (5–46% compared to 0.5–1% in the general population) [90], should our hypothesis be confirmed by well-designed studies, cerebellar tDCS could become an important tool for the treatment of individuals with ASD and comorbid epilepsy.

Nonetheless, this study has several limitations, including a small sample size, the open-label design, and the short-term follow-up, all of which limit the generalizability of the findings. Moreover, recent findings have highlighted the enormous heterogeneity in individual responses to brain stimulation, in part due to baseline activation in the stimulated areas as well as the regional concentration ratio of inhibitory (GABA) and excitatory (glutamate) neurochemicals [91,92]. Because our study does not provide any information about cortical excitability and neurochemical concentrations in the stimulated areas, we cannot rule out that the baseline cortical state of the patients of our small sample might not be representative of the ASD population. In addition, considering the small size of children’s heads, we cannot exclude that brain areas close to the cerebellum and left DLPFC might have been stimulated using our standard-size electrodes, thereby contributing to the observed effects. However, as current density is reduced very rapidly, i.e., by one order of magnitude every 8 mm [93], we can assume that the maximum electrical field in each position was induced in the target region. Finally, because of the design, we cannot exclude the possibility that the expectations of the raters and parents of this novel approach might have influenced their ratings.

Of note, significant heterogeneity in the ABC assessed at baseline contributed to large confidence intervals around the mean pre- and post-tDCS differences. Even though every participant showed improvements, the large confidence intervals are likely to be problematic and need to be addressed when planning a larger study. To address all these issues in a randomized controlled trial, a greater number of participants is needed, perhaps with less heterogeneity in the severity and quality of symptomology. In addition, participants needing psychotropic drugs should be excluded, and methods for standardizing raw scale scores might be applied.

More research is needed in brain modelling, neuroimaging, and neurophysiology aimed at understanding the distinctive mechanisms involved in the effects of this new right cerebellar/left frontal tDCS montage among individuals with ASD. In particular, the dissection of the specific contribution of the two stimulated regions could be assessed by a study consisting of tests for cognitive processes typically subserved by the left DLPFC, such as attention and working memory.

However, whatever the mechanisms involved, it is possible to expect that in highly neuroplastic brains, such as the brains of children with ASD, tDCS has the potential to change the course of the disorder by influencing cell migration via changes in transcription and translation as well as post-translational changes in genes involved in neurodevelopment. Changes such as these could reverse abnormal trajectories of neurodevelopment and prevent cognitive and behavioral symptoms from becoming chronic in nature.

## 5. Conclusions

This pilot study suggests that right cerebellar/left frontal tDCS is feasible, safe, and potentially effective for improving ASD symptoms among children. tDCS was well tolerated, and no serious adverse events were reported. Participants attended all the tDCS sessions as scheduled. All participants demonstrated significant improvements, including unexpected improvements in comorbid epilepsy and tic disorder. Nonetheless, given the paucity of research in this area, it is not yet possible to draw definitive conclusions about the effectiveness and safety of this intervention as well as potential mechanisms of action.

## Figures and Tables

**Table 1:** Demographic and clinical characteristics of participants. OT, occupational therapy; MAT, multisystem aquatic therapy; ST, speech therapy; ABA, applied behavioral analysis.

Subjects	Age	Sex	Handedness	Intellectual Disability	Medical Comorbidities	Current Medication ^a^	Current Behavioral Therapy
1	12	M	Right	-	Tic disorder	Sertraline 100 mg	-
2	13	F	Right	Severe	-	-	MAT ^g^; ABA ^g^
3	11	M	Right	Mild	-	-	ST ^c^; MAT ^c^; ABA ^d^
4	11	M	Right	Moderate	-	-	MAT ^c^
5	11	M	Right	Moderate	-	-	MAT ^b^; ABA ^f^
6	11	M	Right	Moderate	-	Haloperidol 1 mg; Risperidone 5 mg	OT ^e^; MTA ^c^
7	9	M	Right	-	Epilepsy	-	ST ^e^; ABA ^f^

^a^ daily dosage; ^b^ 1 h a week; ^c^ 2 h a week; ^d^ 4 h a week; ^e^ 6 h a week; ^f^ 10 h a week; ^g^ 12 h a week.

## Data Availability

The data presented in this study are available on request from the corresponding author. The data are not publicly available due to privacy restrictions.
